# Surgical outcomes of full endoscopic spinal surgery for lumbar disc herniation over a 10-year period: A retrospective study

**DOI:** 10.1371/journal.pone.0241494

**Published:** 2020-11-05

**Authors:** Chien-Min Chen, Li-Wei Sun, Chun Tseng, Ying-Chieh Chen, Guan-Chyuan Wang

**Affiliations:** 1 Division of Neurosurgery, Department of Surgery, Changhua Christian Hospital, Changhua, Taiwan; 2 School of Medicine, Kaohsiung Medical University, Kaohsiung, Taiwan; 3 College of Nursing and Health Sciences, Dayeh University, Changhua, Taiwan; 4 Department of Orthopaedic Surgery, China Medical University Beigang Hospital, Yunlin County, Taiwan; 5 Department of Neurosurgery, Hualien Tzu Chi Hospital, Buddhist Tzu Chi Medical Foundation, Hualien, Taiwan; Mayo Clinic Minnesota, UNITED STATES

## Abstract

**Objective:**

Full endoscopic lumbar discectomy (FELD) for lumbar disc herniation (LDH) has become popular in recent years. Previous studies have proven the efficacy, but few have discussed the possible risk factors of poor outcome. In this study, we reviewed patients who underwent FELD at Changhua Christian Hospital in the past 10 years and sought to identify factors associated with poor surgical outcomes and re-operations.

**Methods:**

We retrospectively reviewed records from mid-2009 to mid-2018. Patients had undergone FELD and follow-up for ≥1 year were included. Factors included in the outcome evaluations were age, sex, surgical time, body mass index, surgical methods, disc herniation type, extension of herniation, degree of canal compromised, disc degenerative grade, smoking and alcohol use, surgical lumbar level, symptom duration, Oswestry low back disability index, and visual analog scale score. We had evolved from inside-out methods to outside-in methods after 2016, thus, we included this factor in the analysis. The primary outcomes of interest were poor/fair MacNab score and re-operation.

**Results:**

From mid-2009 to mid-2018, 521 patients met our criteria and were analyzed. The median follow-up was 1685 days (range, 523–3923 days). Thirty-one (6.0%) patients had poor surgical outcomes (fair/poor MacNab score) and 45 (8.6%) patients required re-operation. Prolapsed herniated disc (*P* < 0.001), higher disc degenerative grade (*P* = 0.047), higher lumbar level (*P* = 0.026), longer preoperative symptoms (*P* < 0.001), and surgery before 2017 (outside-in technique, *P* = 0.020) were significant factors associated with poor outcomes in univariate analyses. In multivariate analyses, prolapsed herniated disc (*P* < 0.001), higher disc degenerative grade (*P* = 0.030), and higher lumbar level (*P* = 0.046) were statistically significant. The most common adverse symptom was numbness. Factors possibly associated with higher re-operation rate were older age (*P* = 0.045), alcohol use (*P* = 0.073) and higher lumbar level (*P* = 0.069). Only alcohol use showed statistically significant re-operation rates in multivariate analyses (*P* = 0.035).

**Conclusions:**

For treating LDH by FELD, we concluded that prolapsed disc, higher disc degenerative grade, higher lumbar level, and longer preoperative symptom duration were possibly associated with unsatisfactory surgical outcomes (poor/fair MacNab score). The outside-in technique might be superior to the inside-out technique. Older age and alcohol use might be associated with a higher re-operation rate.

## Introduction

Lumbar disc herniation (LDH) causes low back pain, claudication, or sciatica, which might ultimately lead to significant disability [1]. Previously, microsurgery or open surgery was used to treat LDH; however, with advances in surgical instruments and techniques, full endoscopic lumbar discectomy (FELD) has gained popularity. Many studies have proven its benefits, including less blood loss, less wound pain, and shorter recovery time [2–6]. However, large studies discussing potential factors associated with poor surgical outcomes were few [7–12]. Some studies have identified factors, such as herniation type, patient comorbidity, and age, as potential causes of surgical failure or unfavorable outcomes in traditional open or microscopic surgery, but few studies have investigated factors of FELD associating poor outcomes [8,13–19].

FELD has been performed at Changhua Christian Hospital (CCH) for >10 years, and most of our patients had good quality follow-up records. In this study, we retrospectively reviewed our patients who had LDH and underwent FELD at CCH with the aim of identifying factors associated with poor surgical outcomes or re-operations in LDH patients treated by FELD.

## Methods and methods

### Patient selection

We retrospectively reviewed charts and surgical records from mid-2009 to mid-2018. Patients who had undergone FELD and follow-up for ≥1 year were included. Patients who were lost to follow-up or had diseases other than LDH were excluded. The study protocol was approved by the ethics review board of Changhua Christian Hospital, Taiwan (IRB No.190905). All surgeries were performed by the first author. All the patients’ ID and name were fully anonymized when conducting the analysis. the data was collected and accessed on 2019/12.

#### Outcomes evaluated

**Fig 1:**
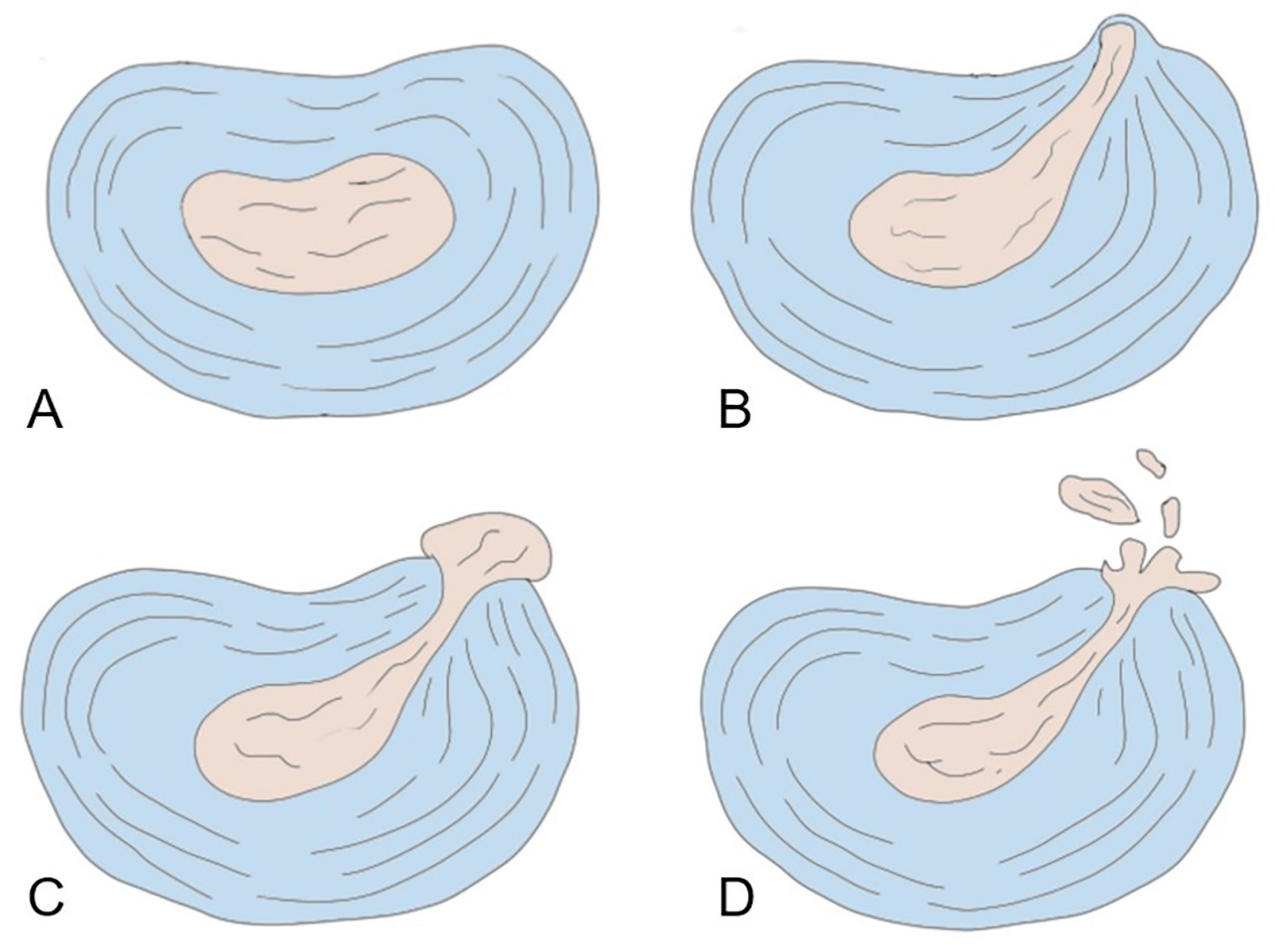
Disc herniation type. A: Normal. B: Prolapsed. C: Extrusion. D: Sequestration.

**Fig 2:**
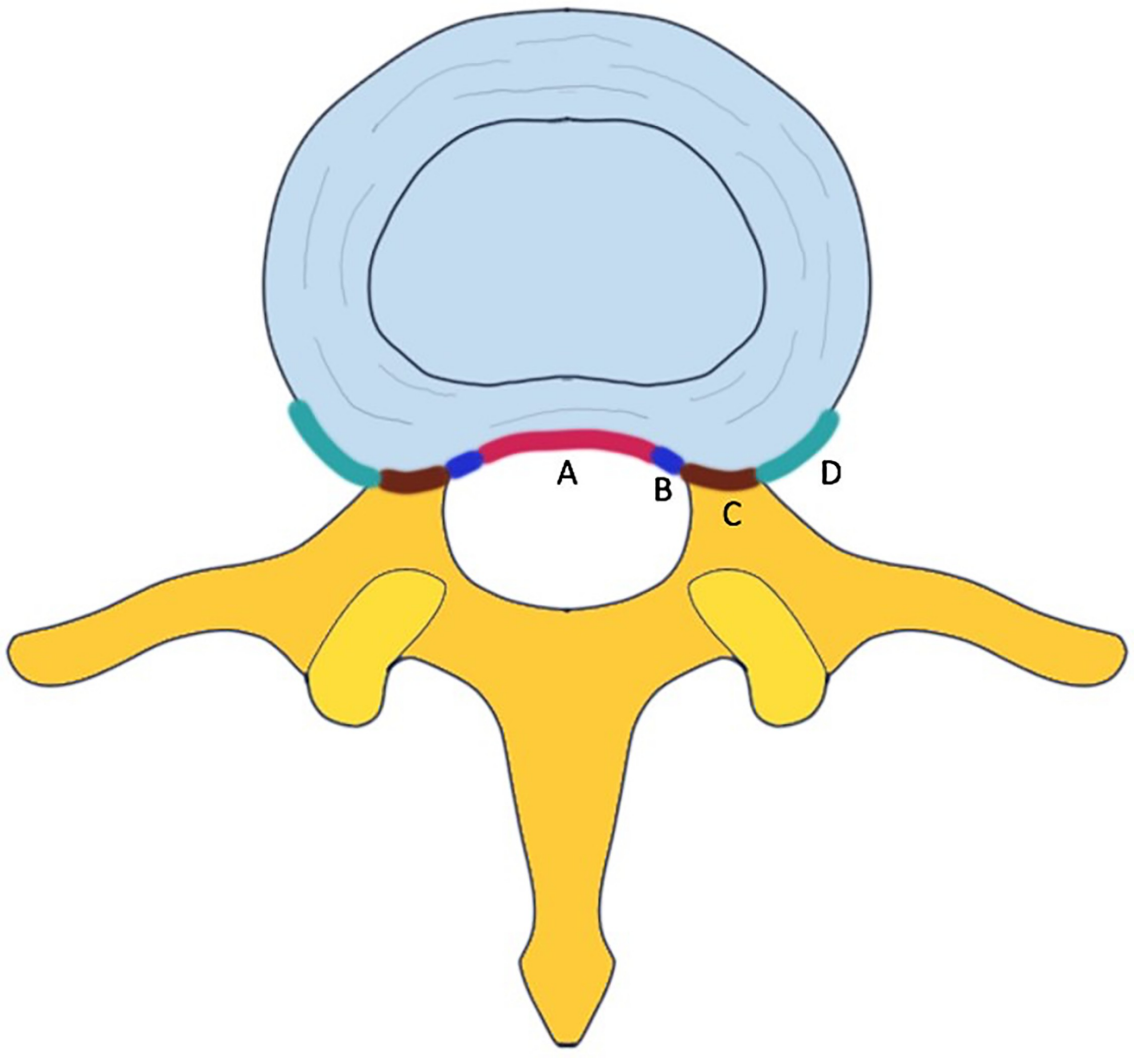
Extension of herniated disc. A. Central type. B. Subarticular type. C. Foraminal type. D. Extraforaminal type.

**Fig 3:**
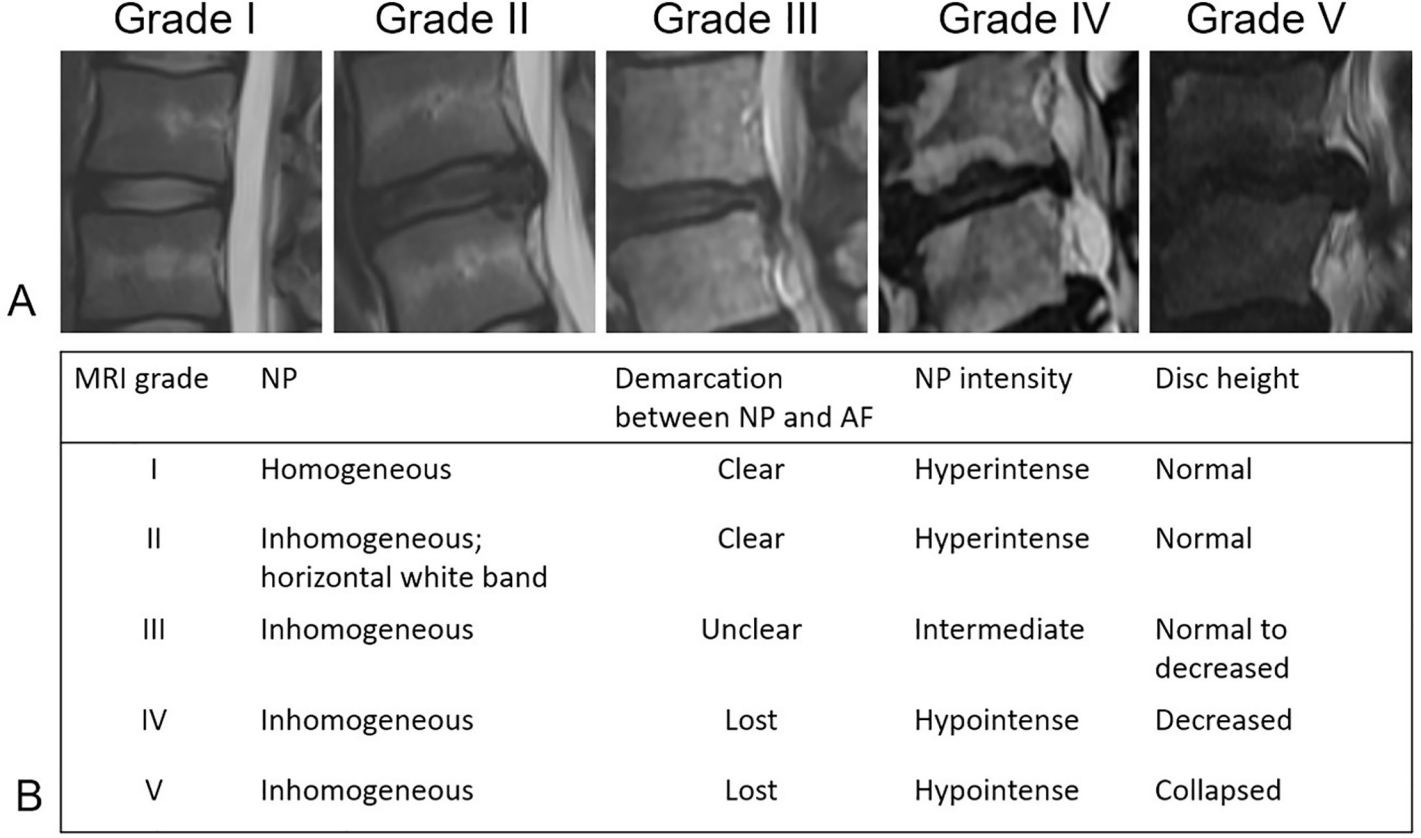
Disc degenerative grading from grade I to grade V. A: Disc degenerative grading by T2-weighted MRI. B: Disc degeneration graded by homogeneity of the nucleus pulposus, demarcation between the nucleus pulposus and annulus fibrosus, intensity of the nucleus pulposus, and disc height. NP: Nucleus pulposus. AF: Annulus fibrosus.

Factors included in the outcome analysis were age, sex, surgical time, body mass index (BMI), surgical method (transforaminal or interlaminar), disc herniation type (Fig 1), extension of the herniation (Fig 2), >50% canal compromised or not, disc degenerative grade (Fig 3), surgical level (L4/L5 or L5/S1 versus other lumbar levels), smoking, alcohol use, symptom duration, age, Oswestry low back disability index (ODI), visual analog scale (VAS), and year of surgery (before 2016 versus after 2017).

We routinely used the ODI, VAS, and MacNab scores to evaluate the surgical outcomes and patients’ satisfaction at 3months, 6months and 12 months. In this study, the preoperative score was recorded. The postoperative score was recorded at the last time the patients visited the clinic or in December 2019 when we followed up the patients by telephone call. The ODI was used to evaluate the outcome of low back pain, and the higher the score, the worse the quality of life and disability [20] (range, 0%–100%). The VAS is a unidimensional scale used to measure a patient’s pain based on the patient’s subjective feeling [21]; the higher the score, the higher the intensity of pain (range, 0–10). The MacNab criteria were useful for evaluating the patients’ satisfaction with surgery and were classified as excellent, good, fair, and poor [22]. Fair and poor MacNab scores were usually considered to be unsatisfactory outcomes [6,23,24]. Re-operation was defined as the need for a patient to undergo a second surgery at the same level because of prior surgical failure or recurrence. The patients had remitted symptoms and the images were compatible with the symptoms at the same surgical level. In this study, the MacNab score (good/excellent vs. fair/poor) and re-operation rate were the primary outcomes.

Symptom duration was defined as the time from initial symptoms to the operation day. Surgical time was the duration of the operation and was classified as >1 hour and ≤1 hour. The BMI was calculated by dividing the patient’s weight in kilograms (kg) by the square of the height in meters. We divided the BMI into ≥27 and <27 since 27 is the cutoff value for defining “obese” in Taiwan.

The disc herniation type, extension of herniation, degree of spinal canal compromised, and disc degenerative grading were interpreted from preoperative magnetic resonance imaging (MRI). The disc herniation type was categorized into prolapsed, extrusion, and sequestration (Fig 1) [8]. The extension of herniation was classified into central, subarticular, foraminal, and extraforaminal (Fig 2). Our interpretation of disc degenerative grading was based on Fig 3 [25].

We divided the year of surgery at 2016 because we used the inside-out technique before 2016 but evolved to the outside-in technique after 2017. There transforaminal approach has two primary concepts: the inside-out and outside-in techniques [3,26,27]. The inside-out technique is a transforaminal approach that docks the working channel inside the disc and removes the herniated disc. However, the outside-in technique anchors the working channel (SPINENDOS GmbH, Munich, Germany) to the facet joint first, performs foraminoplasty and then advances the working channel to find the herniated disc and remove it. We evaluated whether these two surgical techniques result in different outcome. In this analysis, we only included patients who received transforaminal approach and did not mix it with multivariate analyses.

#### Statistical analysis

The chi-square test was applied to compare categorical variables. The binary logistic regression was applied to multivariate analysis. A *P* value of <0.05 was considered to be indicative of statistical significance. IBM SPSS 22 (IBM Corporation, Armonk, NY, USA) was used as the statistical analysis software.

## Results

From mid-2009 to mid-2018,521 patients met our criteria and were analyzed (there were totally 582 patients underwent FELD for LDH but 61 patients had missing data or lost follow-up). The median follow-up was 1685 days (range, 523–3923 days). Thirty-one (6.0%) patients had poor surgical outcomes (fair/poor MacNab score) and 45 (8.6%) required re-operation. The number of all adverse outcomes, including fair/poor MacNab scores and re-operations, was 63 (12.1%). Numbness was the most common adverse postoperative symptom followed by pain. After the surgery, the mean VAS score improved from 7.3 to 0.3, and the mean ODI score improved from 26.9 to 2.7. The patients’ demographics are detailed in Table 1.

**Table 1:** Patient characteristics.

	Patient numbers (%)
Total number of patients	521
Sex	
Male	331 (63.5%)
Female	190 (36.5%)
Operative methods	
Transforaminal	319 (61.2%)
Interlaminar	202 (38.8%)
Re-operation patients	45 (8.6%)
MacNab score	
Excellent/good	490 (94%)
Fair/poor	31 (6%
All adverse outcome*	63 (12.1%)
Adverse symptoms**	
Numbness	20
Pain	14
Weakness	2
Soreness	4
Numbness+pain	7
Numbness+weakness	4
Numbness+soreness	2
Dysethesia	2

*All adverse outcome was defined as fair/poor MacNab score and re-operation.

**Post-operative adverse symptoms.

The analysis showed that prolapsed herniated disc (prolapse type:22.2% vs. extrution type:4.0% vs sequestration type:5.8%,*P* < 0.001), higher disc degenerative grade (grade 4:13.3% vs grade 3:7.1% vs. grade 2:2.1%,*P* = 0.047), higher lumbar level (L4-S1:5.0% vs. L1-L4:11.4%,*P* = 0.026), longer preoperative symptoms (>1year:13.7% vs. < = 1year:5.2%,*P* < 0.001), and surgery before 2017 (< = 2016 inside-out:7.4% vs. >2017 outside-in technique:2.1%,*P* = 0.020) were significant factors associated with poor outcomes in univariate analyses. In multivariate analyses, prolapsed herniated disc (*P* < 0.001), higher disc degenerative grade (*P* = 0.030), and higher lumbar levels (p = 0.046) were associated with poor surgical outcomes (Table 2).

**Table 2:** Factors related to fair/poor MacNab score.

	Univariable analysis		Multivariable analysis	
Variable	*Rate of fair/poor MacNab score*	*P*	*Hazard ratio*	*P*
Age		0.078		
< = 60 y/o	4.9%			
>60 y/o	9.1%			
Sex		0.300		
Male	5.1%			
Female	7.4%			
Surgical time		0.154		
< = 1hr	4.6%			
>1hr	7.6%			
BMI		0.442		
<27	6.5%			
>27	4.8%			
Surgical methods		0.127		
Transforaminal	7.2%			
Interlaminar	4.0%			
Disc herniation type		<0.001		<0.001
Prolapse	22.2%		1	
Extrusion	4.0%		0.129 (0.047–0.358)	
Sequestration	5.8%		0.193 (0.053–0.509)	
Extension of herniation		0.236		
Central	7.5%			
Subarticular	3.1%			
Foraminal	5.0%			
Extraforaminal	9.4%			
>50% canal compromised		0.164		
No	5.2%			
Yes	9.2%			
Disc degenerative grade				0.030
2	2.1%		1	
3	7.1%	0.047	4.161 (1.176–14.721)	
4	13.3%		12.029 (1.672–86.567)	
Smoking		0.167		
No	5.8%			
Yes	6.9%			
Alcohol		0.113		
No	5.5%			
Yes	11.4%			
Surgical level		0.026		0.046
L4-S1	5.0%		1	
L1-L4	11.4%		2.516 (1.108–6.216)	
Symptom duration		<0.001		0.118
< = 1 year	5.2%		1	
>1 year	13.7%		2.277 (0.811–6.391)	
Pre-op ODI		0.234		
< = 30	5.1%			
>30	7.7%			
Pre-op VAS		0.397		
< = 6	7.1%			
>6	5.3%			
Year of surgery**		0.020		
< = 2016	7.4%			
>2017	2.1%			

*only included patient who underwent transforaminal approach so year of surgery was not included in the final multivariable analysis.

Potential risk factors toward causing higher re-operation rate were age (≤60 years:7.2% vs. >60 years:12.9%, *P* = 0.045), alcohol consumption (alcohol consumption:15.9% vs. no alcohol consumption: 8.0%, *P* = 0.073) and higher lumbar level (L4-S1: 7.7% vs. L1-L4: 11.4%, *P* = 0.069) (Table 3). We included these factors in the multivariate analyses but found out that only alcohol consumption was statistically significant resulting in higher re-operation risk (*P* = 0.035) (Table 3).

**Table 3:** Factors related to re-operation rate.

	Univariable analysis		Multivariable analysis	
Variable	Re-operation rate	*P*	*Hazard ratio*	*P*
Age		0.045		0.07
< = 60 y/o	7.2%		1	
>60 y/o	12.9%		1.896 (0.950–3.782)	
Sex		0.153		
Male	10.0%			
Female	6.3%			
Surgical time		0.164		
< = 1hr	7.1%			
>1hr	10.5%			
BMI		0.887		
<27	8.8%			
>27	8.4%			
Surgical methods		0.433		
Transforaminal	9.4%			
Interlaminar	7.4%			
Disc herniation type		0.549		
Prolapse	11.1%			
Extrusion	7.6%			
Sequestration	10.3%			
Extension of herniation		0.745		
Central	8.8%			
Subarticular	7.5%			
Foraminal	11.0%			
Extraforaminal	6.3%			
>50% canal compromised		0.530		
No	8.3%			
Yes	10.5%			
Disc degenerative grade		0.473		
2	10.6%			
3	7.7%			
4	13.3%			
Smoking		0.534		
No	8.3%			
Yes	10.3%			
Alcohol		0.073		0.035
No	8.0%		1	
Yes	15.9%		2.632 (1.069–6.483)	
Surgical level		0.069		0.222
L4-S1	7.7%		1	
L1-L4	13.9%		1.617 (0.747–3.498)	
Symptom duration		0.186		
< = 1 year	8.2%			
>1 year	13.7%			
Pre-op ODI		0.870		
< = 30	8.5%			
>30	8.9%			
Pre-op VAS		0.352		
< = 6	10.1%			
>6	7.7%			
Year of surgery		0.587		
< = 2016	10.0%			
>2017*	8.2%			

*only included patient who underwent transforaminal approach.

## Discussion

Many investigators have reported possible risk factors of failed spinal surgery, but we focused on FELD for LDH in this study. We did not enroll spinal fusion or pure spinal stenosis cases because we considered them to be a different topic. The reasons were (1) different surgical indications (e.g., unstable lumbar spine or high-grade spondylolisthesis), (2) more invasive (the need for total to subtotal discectomy and cage implantation), and (3) different reasons for surgical failure (cage dislodgement or subsidence).

Previous studies have found that some factors might influence surgical outcomes and re-operation rate for LDH, but few studies have examined outcomes of FELD. In addition, to the best of our knowledge, our study had the largest number of patients and longest follow-up for analysis of outcomes of LDH treated by FELD. Besides, all of the operations were performed by one surgeon which eliminated the variable causing by different surgeon’s experience and technique.

### Patient factors versus outcome

Previous research has shown that the preoperative status of some patients was a potential risk factor for poor outcome or recurrence [8,15–18,23]. Older age has been shown to be a risk factor for poor outcome or recurrence in many studies [7,15,18,23,28]. In the present study, older age was significantly associated with poorer surgical outcome but not with recurrence rate in univariable study. Even in multivariable study, it showed a trend toward higher risk of poor outcome (HR:1.895, *P* = 0.07) (Table 2). The comorbidities, poor compliance, and poor rehabilitation of some older patients might explain this result.

Sex has not usually been considered to be a potential risk factor in LDH [18,24,29,30]. In our study, male did not show statically significant in surgical outcome and re-operation rate.

Smoking has been shown to be a cause of poorer surgical outcome and higher recurrence in spinal surgery [8,31–33]. For alcohol, the results were more complicated. Our patients were asked how often they drank alcohol in a week. If they consumed alcohol beverages more than 4 days a week, then we recorded the patients to had alcoholic habit. Some studies have shown that alcohol was associated with poorer outcome, but other studies have shown that drinking wine could improve outcomes after lumbar discectomy [34–36]. We found that alcoholic patients had a higher risk of recurrence than non-alcoholic patients in multivariable analysis (HR:2.632,*P* = 0.035), but not associated with poor surgical outcome. Our assumption was that alcohol related to pure bone density and induced osteopenia, osteoporosis and biomechanically instability [37]. The decreased weight bearing ability of the vertebral body wound translate the force to facet joint or disc which lead to spondylosis or herniated disc. Smoking failed to show any effect on surgical outcome or re-operation rate in our results.

Longer duration of symptoms has been shown to be associated with unfavorable prognosis [16,17,24,38,39]. In our study, symptoms duration more than 1 year was associated with poorer surgical outcome in univariable analysis (13.7% vs 5.2%, *P*<0.001) but not in multivariable analysis (HR:2.277,*P* = 0.118). The re-operation rate was higher when symptoms duration more than 1 year (13.7% vs 8.2%) but did not show statically significant (*P* = 0.186). We assumed that longer symptoms duration might lead to hard disc, epidural venous congestion or fibrotic adhesions which increased the surgical difficulty and resulted in poorer surgical outcome.

Some studies have shown that higher preoperative ODI and VAS scores were associated with poorer outcome, but some have not [16,17,24,40]. Our study did not show that higher preoperative ODI and VAS were associated with surgical outcome or re-operation. However, the VAS and ODI scores were obtained from self-evaluated questionnaires, so substantial variations between study groups could exist. This might explain this discrepancy between our results and those from previous studies.

Initially, we assumed that patients with obesity (BMI > 27) would have poorer surgical outcome and higher recurrence, but the results showed that BMI did not significantly affect either. However, some studies have shown higher BMI to be an adverse prognostic factor associated with higher recurrence [19,29,40].

### Surgical method versus outcome

We did not find whether transforaminal or interlaminar approach affected the surgical outcome or re-operation rate. However, we further investigated whether different transforaminal approach resulted in different outcomes. There transforaminal approach has two primary concepts: the inside-out and outside-in techniques [3,26,27]. There is no reliable evidence that the outside-in was superior to the inside-out technique in terms of outcomes in previous studies [27]. However, because the inside-out technique has a limited surgical field and outside-in technique has a wider surgical field because of foraminoplasty, there might be more remaining disc or incomplete decompression in the inside-out technique [41]. We changed our surgical methods from inside-out to outside-in since 2017, thus, we divided the surgical time at this point in the analysis. We found that surgery performed after 2016 were associated with better surgical outcomes (7.4% vs. 2.1%, *P* = 0.020) but the re-operation rate was similar (10.0% vs. 8.2%, *P* = 0.587). The foraminoplasty in the outside-in technique might help in root decompression and more working space and visualization for disc retrieval. The limitation was that there were no clear cut distinction between the transformation of the surgical methods; although we used 2017 as the cut-off point, there might be some overlapping of the two surgical methods.

Initially, we assumed that longer surgical time might result in poorer outcome because longer surgical time means that the surgery was more difficult and that there was a possibly longer traction time for the nerve root. However, we did not find a significant association between longer surgical time and surgical outcome and re-operation rate.

### Disc type versus outcome

For the type of herniated disc, we found that the prolapse type had the worse outcome in univariable and multivariable analysis, which was similar to the results of previous studies [8]. In the univariable analysis, the prolapse type of disc resulted in 22.2% poor surgical outcome (*P*<0.001) and had the hazard ratio 7.752 and 5.181 compared to extrusion and sequestration type of disc (*P*<0.001). Carragee et al. showed that the fragment-containing (also considered to be a prolapsed type) group had the worst outcome in terms of symptoms improvement after lumbar discectomy and the sequestration type had the best outcome [14]. Dewing et al. conducted a prospective clinical study that showed that young patients with contained disc herniation (also considered to be a prolapsed type) had a worse prognosis than those of the other two groups [42]. We had two assumptions. First, a prolapsed disc might not be the cause of low back pain or radiculopathy in some patients. A patient’s lower back pain might be misdiagnosed as the result of a prolapsed disc on MRI; therefore, surgery would have little benefit. On the contrary, extrusion and sequestration are usually the causes of symptoms. Second, we found that patients with prolapsed discs had longer symptom duration before the surgery (prolapsed disc: 456.8 days vs. other types: 182.2 days, *P* < 0.001), which was the cause of unfavorable outcome in our study.

Disc degeneration grade based on preoperative MRI has been shown to be a prognostic predictor in lumbar spinal fusion [29,30,43]. A study in 2019 showed that vacuum disc, loss of disc height > 3 mm, and advanced degeneration were associated with poor surgical outcome in endoscopic transforaminal surgery [28]. We used the Pfirrmann system and found that higher grade was associated with poorer surgical outcome in both univariate and multivariate analyses but not with re-operation rate. Disc degenerative grade 4 had 13.3% poor surgical outcome compared to 7.1% in grade 3 and 2.1% in grade 2 (p = 0.047). The poorer surgical outcome might be explained by the higher disc degeneration grade; especially, a substantial decrease in disc height has been found to be associated with instability, and simple discectomy or decompression was insufficient [28]. Spinal fusion to restore the disc height and spinal stability might be a more appropriate choice.

Yong Ahn et al. published a paper in 2018 showing that intracanal disc herniation, which included central and subarticular types, was associated with better prognosis than those of other types [23]. Central disc herniation has also been shown to be associated with recurrence in FES [19]. We did not show the extension of disc herniation was associated with surgical outcome and re-operation rate. The present study population was chosen to focus on the transforaminal approach and lumbar disc herniation, although we did not limit our patients to these two criteria, which might explain the discrepancy in the results.

Our study showed that the herniated disc at the upper lumbar level (L1-L4) had poorer surgical outcome in univariate (11.4% vs. 5.0%, *P* = 0.026) and multivariate analyses (HR:2.516, *P* = 0.046). The re-operation rate was also higher in the upper lumbar level (13.9% vs. 7.7%) but did not reach statistical significance (*P* = 0.069). Studies have shown that upper LDH was associated with higher re-operation rate [19] but did not influence surgical outcomes [7]. We assumed that the relative wide interlaminar space of L4/L5 and L5/S1 reduced the surgical difficulty. However, the upper lumbar level had narrower interlaminar space, presence of conus medullaris, and anatomical obstacles interfering with the endoscopic route (eg., ribs and kidney might limit the transforaminal trajectory).

Overall, we found that more factors were related to poorer surgical outcome than to re-operation rate. We assumed that re-operation rate was more dependent on patients’ underlying factors (eg., alcoholism and age). With an experienced surgeon, the re-operation rate was not affected by disc type, surgical methods, surgical level, or duration or symptoms.

### Study limitations

There were some limitations in this study. First, although all of the surgeries were performed by the same surgeon, we did not count on the learning curve. Surgeries performed in the earlier years might have had poorer outcomes, which might have affected the results. Second, the patients’ living habits and working styles might also have influenced the outcomes. For example, patients whose work involved lifting heavy weights or frequent bending might have had a higher recurrence rate. Since it was difficult to standardize the assessment of the patients’ workloads and living habits (e.g., how much bending and heavy weight lifting were performed in a day), we did not include that assessment in the analysis. Third, we used poor and fair MacNab scores to define a poor outcome, and some studies have used the postoperative ODI or VAS score. This difference in definitions of outcomes might have led to different results. Last, though we showed alcohol was the solely factors related to re-operation rate in multivariable analysis, we did not detail how much and what kind of alcohol the patients consumed. The threshold of alcohol consumption leading to recurrent LDH might need further investigation.

## Conclusion

For treating LDH by FELD, we concluded that prolapse type herniation, higher disc degenerative grade, higher lumbar level were the main factors associated with unsatisfactory surgical outcomes (poor/fair MacNab scores). The outside-in technique had more optimal surgical results than the inside-out technique. Only alcoholic use was associated with higher re-operation rate. Knowing these potential risk factors for FELD might help surgeons to preoperatively identify patients at high risk of poor outcomes.
